# SolexaQA: At-a-glance quality assessment of Illumina second-generation sequencing data

**DOI:** 10.1186/1471-2105-11-485

**Published:** 2010-09-27

**Authors:** Murray P Cox, Daniel A Peterson, Patrick J Biggs

**Affiliations:** 1 Institute of Molecular BioSciences, Massey University, Palmerston North 4442, New Zealand; 2 The Allan Wilson Centre for Molecular Ecology and Evolution, New Zealand; 3 The Bio-Protection Research Centre, New Zealand; 4 Institute of Veterinary, Animal and Biomedical Sciences, Massey University, Palmerston North 4442, New Zealand; 5 Massey Genome Service, Massey University, Palmerston North 4442, New Zealand

## Abstract

**Background:**

Illumina's second-generation sequencing platform is playing an increasingly prominent role in modern DNA and RNA sequencing efforts. However, rapid, simple, standardized and independent measures of run quality are currently lacking, as are tools to process sequences for use in downstream applications based on read-level quality data.

**Results:**

We present SolexaQA, a user-friendly software package designed to generate detailed statistics and at-a-glance graphics of sequence data quality both quickly and in an automated fashion. This package contains associated software to trim sequences dynamically using the quality scores of bases within individual reads.

**Conclusion:**

The SolexaQA package produces standardized outputs within minutes, thus facilitating ready comparison between flow cell lanes and machine runs, as well as providing immediate diagnostic information to guide the manipulation of sequence data for downstream analyses.

## Background

Second-generation technologies are rapidly coming to dominate modern DNA and RNA sequencing efforts [1]. Among the available systems, Illumina sequencing (known informally as Solexa) is playing an increasingly prominent role. However, the error profiles of high-throughput short read sequencing technologies differ markedly from traditional Sanger sequencing [2]; they tend to exhibit a steep, exponential increase in error rates along the read length, and are susceptible to a wider range of chemistry and machine failures (such as air bubbles in system fluidics). Although the quality of second-generation sequencing data affects downstream applications, monitoring and diagnosis of data quality has not kept pace with the rapid rate of improvement seen in other aspects of the technology.

Owners of Illumina machines have access to on-board diagnostic tools, which give detailed information about data quality for each lane, tile and nucleotide position. However, these tools are not available to most users, the majority of whom now outsource data collection to dedicated sequencing centers. In our experience, these centers do not usually release data quality information, although we advocate strongly that they should. Lacking this information, users must turn to publicly available software packages to quantify data quality. The R package TileQC [3], which offers similar functionality to Illumina's proprietary software, can help identify some problems at the level of tiles (*e.g*., air bubbles), and in many cases, can even track variation at individual read positions. However, the underlying algorithm relies on errors determined from read mapping, thus requiring a reference genome sequence. TileQC is less useful for the many sequencing projects now being performed on non-model organisms. Several other software packages offer similar functionality for assessing data quality [4,5], but seldom in a quick, automated way that can easily be run by users with limited bioinformatics skills and/or computer resources.

In complementary fashion, software has been written to help correct sequences containing some of these errors, such as image boundary effects [6] - at least for earlier versions of the Illumina technology. However, the ever-increasing quantity of data produced by Illumina sequencers seldom makes such detailed analysis of individual tiles feasible, or indeed, a cost effective use of expensive (and often limited) bioinformatics resources. Nevertheless, major quality defects, particularly failures of entire tiles or individual nucleotide positions must still be accommodated in downstream analyses (*i.e*., by exclusion, or preferably, selective trimming of reads). Simple tabular and graphical summaries of run quality are therefore a necessary prerequisite for any downstream analysis.

Here, we present SolexaQA, a user-friendly software package that provides rapid, at-a-glance assessment of read quality for data generated using Illumina's sequencing technology.

## Implementation

Programs, manuals and example datasets for the SolexaQA package can be downloaded from the project website http://solexaqa.sourceforge.net/.

SolexaQA has minimal runtime requirements, but is nevertheless designed primarily for use on the high-performance UNIX machines that are necessary for analyzing Illumina sequence data. SolexaQA is primarily written in Perl, but integrates graphical capability from the statistics package R [7] and the heatmap visualizer matrix2png [8]. By default, the program produces tables summarizing data quality, but R and matrix2png must be installed for proper functioning of the package's graphical features. Note that matrix2png also requires a working installation of the GD graphics library http://www.libgd.org/.

SolexaQA inputs one (or multiple) sequence read files in Solexa- or Illumina-style FASTQ format, which contains information about base calls as well as associated quality scores [9]. We checked whether these quality scores match actual error rates by mapping reads back to a haploid reference sequence that was *de novo *assembled from the same read dataset. We found that the quality scores returned by the Illumina pipeline (version 1.4) are quite accurate, and if anything, slightly conservative.

SolexaQA reads in FASTQ sequence files containing any number of cycles (*i.e*., nucleotide positions) or tiles (*i.e*., subunits of a flow cell lane), including those produced by early versions of the Illumina pipeline, right up to current pipeline version 1.6. The package also accommodates the virtual tiles employed by the latest revisions to Illumina's sequencing technology (*e.g*., the HiSeq 2000).

**Figure 1:**
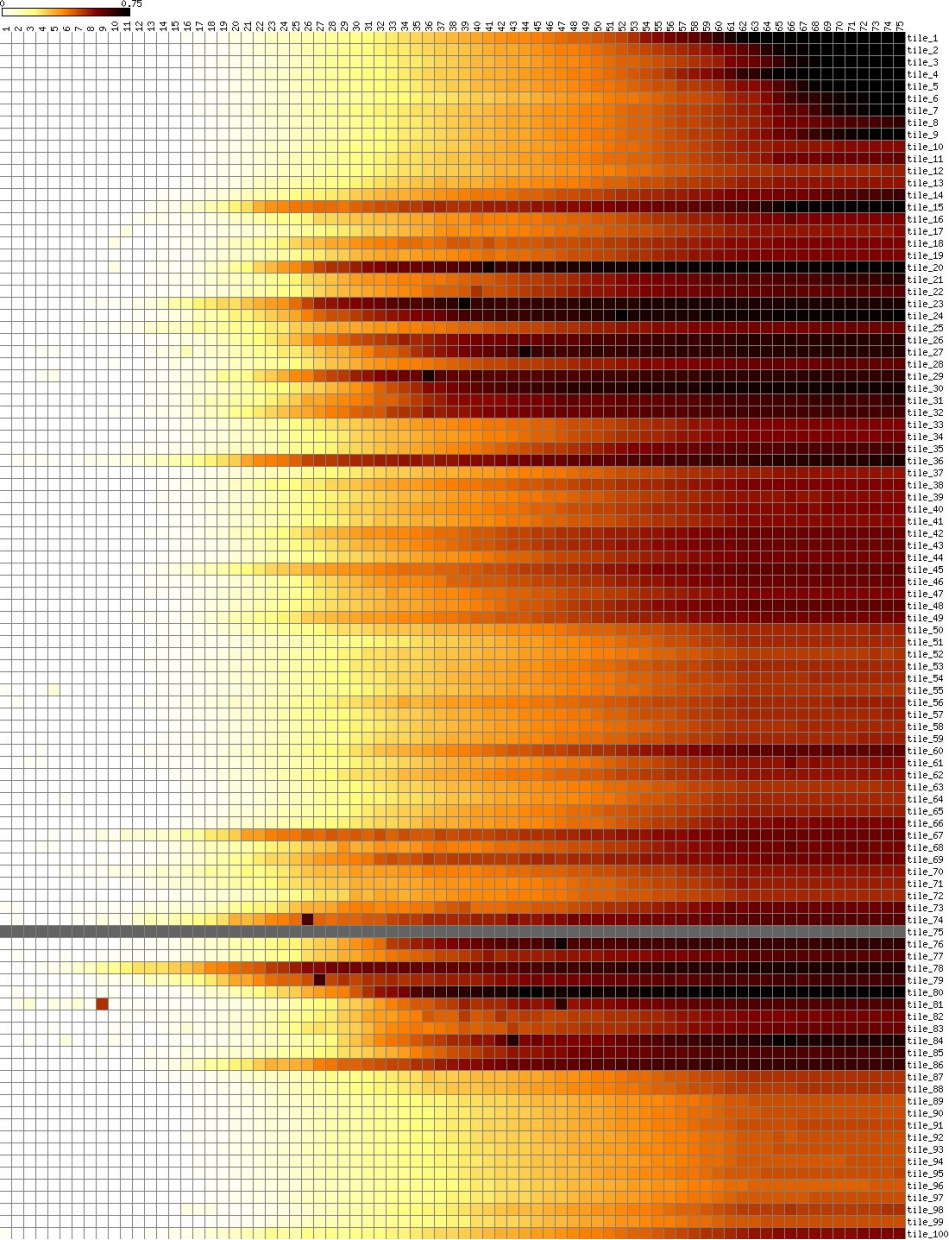
**Example heat map showing several commonly observed quality defects**. Nucleotide positions 1-75 are plotted from left-to-right along the *x*-axis; tiles 1-100 are ranked from top-to-bottom along the *y*-axis. (These numbers may vary for other datasets). The scale depicts the mean probability of observing a base call error for each tile at each nucleotide position. The defects evident in this dataset (see text for details) are atypical of Illumina sequencing; this dataset was chosen specifically to illustrate the capabilities of SolexaQA.

**Figure 2:**
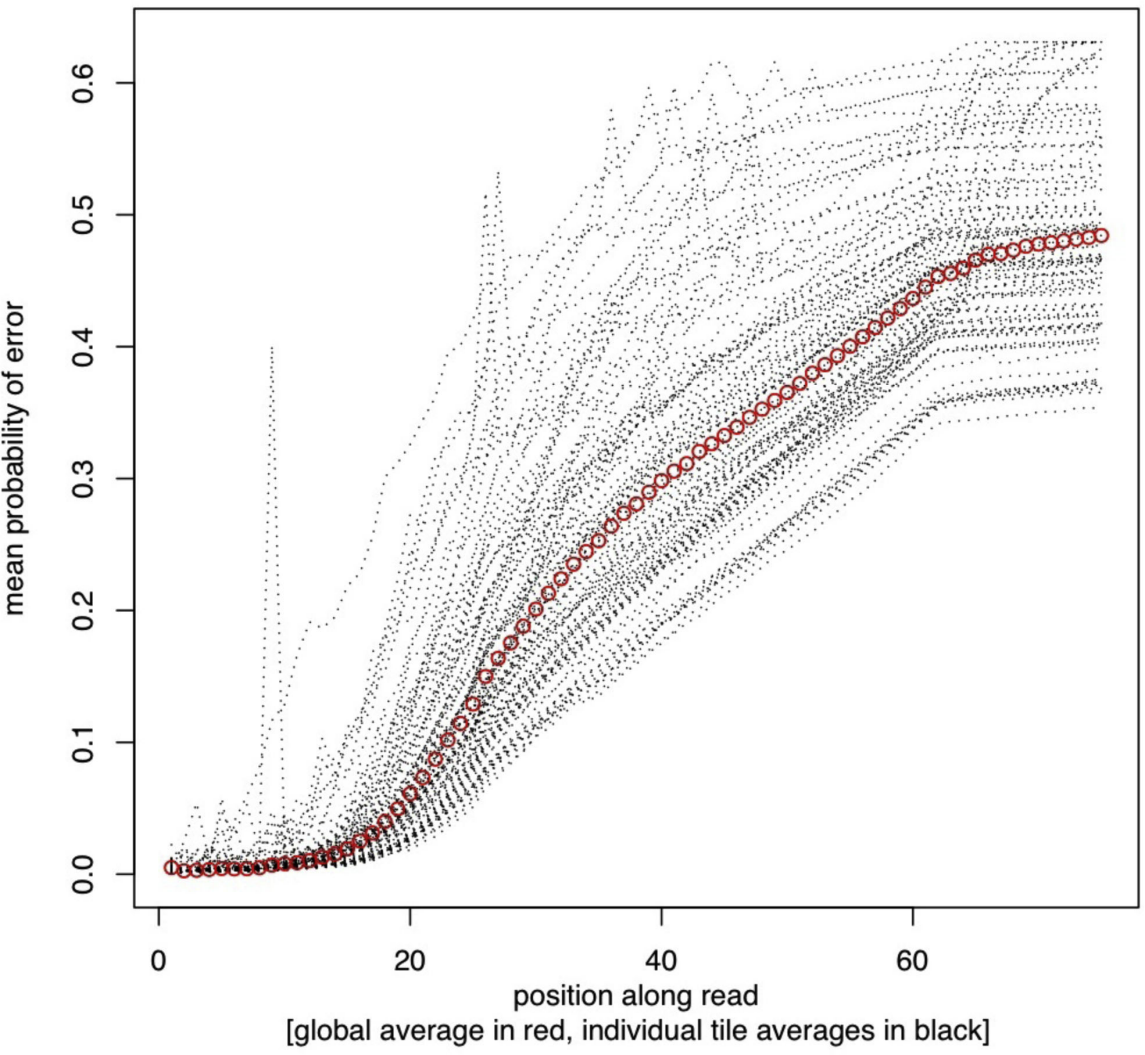
**Distribution of mean quality (probability of error, *y*-axis) at each nucleotide position (*x*-axis) for each tile individually (dotted black lines) and the entire dataset combined (red circles)**. Note the considerable variance in data quality between tiles. The defects evident in this dataset (see text for details) are atypical of Illumina sequencing; this dataset was chosen specifically to illustrate the capabilities of SolexaQA.

SolexaQA calculates a range of summary statistics for a subset of reads drawn randomly from each tile at each nucleotide position; by default, 10,000 reads (typically about 3% of reads at time of writing) are sampled per cycle and tile, but users can tune this parameter via a command line flag. From our observations, we suggest that summary statistics should be calculated from no fewer than 5,000 reads per cycle and tile; the accuracy of statistical calculations begins to erode quickly when fewer reads are sampled. SolexaQA only calculates mean quality scores by default, but users may also request variances, as well as the minimum and maximum quality scores observed. For convenience, the software returns these summary statistics in tabular form. However, SolexaQA also produces graphical displays of mean quality per tile and cycle. This information is presented both as a heat map (Figure 1) and a line graph (Figure 2); the latter also indicates global mean quality for the entire dataset.

**Figure 3:**
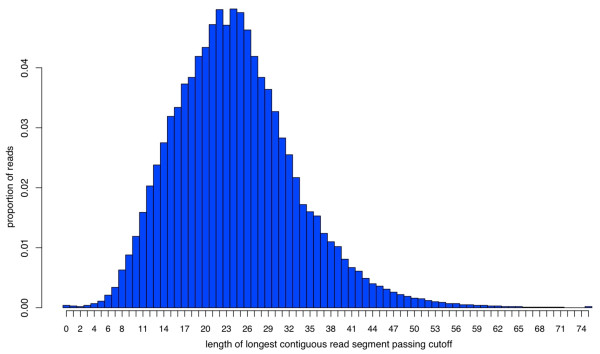
**Distribution of longest read segments passing a user-defined quality threshold (here, *P *= 0.05, or equivalently, Phred quality score *Q *≈ 13, or a base call error rate of 1-in-20)**. Note that reads in this dataset would be trimmed on average to ~25 nucleotides (*i.e.*, only approximately one-third of the initial 75 nucleotide read length). The defects evident in this dataset (see text for details) are atypical of Illumina sequencing; this dataset was chosen specifically to illustrate the capabilities of SolexaQA.

SolexaQA also produces a histogram of maximized read lengths (*i.e*., the distribution of longest contiguous read segments for which base quality scores exceed a user-defined threshold) (Figure 3). Users can select a quality threshold (*i.e*., a Phred quality score, or its associated probability value); otherwise, the software defaults to *P *= 0.05 (or equivalently, *Q *≈ 13, or 1 base call error every 20 nucleotides). This histogram (and associated tabular file) can be considered one representation of the 'usable' information content of a given dataset. For convenience, an additional program, DynamicTrim, has been released as part of the SolexaQA package. This software trims each read to its longest contiguous read segment (from either or both ends) where quality scores exceed a user-defined threshold, and writes this information to a standard Solexa- or Illumina-style FASTQ file [9]. A more detailed discussion of the trimming algorithm is provided online at the project website.

Finally, we note that sequence quality is often described in terms of log probabilities. For instance, *Q *= 30 is the equivalent of *P *= 0.001 (*i.e*., a 1-in-1000 probability of observing an incorrectly called base). This notation is convenient for computational reasons; ASCII characters can readily encode log probabilities rounded to integer values (*e.g*., the character "^" in this particular example). However, although this shortcut is convenient for reducing file sizes, log probabilities are not particularly intuitive. Indeed, some summaries of data quality can even be misleading when calculated as log values (*e.g*., consider the difficulty of interpreting variances or summations of log probabilities). For this reason, the tables and graphs produced by SolexaQA report actual probabilities of error, not log-based quality scores.

## Results and Discussion

### Example dataset

Using default settings (recommended for most users), SolexaQA can process a single FASTQ input file (~4 gigabytes) in under 5 minutes with negligible memory demands on a computer with a fairly standard 2.93 GHz Xeon processor. To illustrate the package's capabilities, we consider the first read of a 75-bp paired-end run generated on the Genome Analyzer II (*i.e*., with 75 cycles and 100 tiles). This example dataset can be represented by a heat map (Figure 1), and illustrates several different types of errors. Firstly, the heat map shows the failure of an entire tile; no reads in tile 75 (Figure 1, grey horizontal bar) passed the quality threshold required by Illumina's pipeline software. Secondly, individual tiles suffered cycle specific failures, as indicated by dark squares in cycles 9, 26 and 27 (Figure 1, lower left). These drops in data quality are often due to tile-specific air bubbles, although they can be caused by other factors as well (*e.g*., oil loss or spills on the Genome Analyzer II series of machines). Finally, tiles on this version of the Illumina platform are arranged in a U-shape: spatially, tiles 1 and 100 are located together at one end of the flow cell, tiles 50 and 51 lie together at the other end, and tiles 25 and 75 fall together in the middle. The clustered association of darkened horizontal lines around tiles 25 and 75 indicates that data quality in this particular run eroded near the middle of the flow cell, but improved towards either end. For some applications (*e.g*., *de novo *read assembly), one or more of these defects may require manipulation of sequence reads. In some instances, these issues may be sufficiently disruptive to require data collection to be repeated. Here, these various data defects are readily apparent after very simple quality analysis using the SolexaQA package. The generally poor quality of this particular dataset, which was chosen solely for didactic purposes, is also captured in graphs that show mean data quality per nucleotide position (Figure 2), as well as the distribution of longest contiguous read segments for which base quality scores have an error rate less than 1-in-20 (Figure 3). Nevertheless, we emphasize that some proportion of good quality data can usually be obtained even from very poor quality runs. Dynamic trimming (described in the following section) is one way to extract these high quality reads. Finally, we note that we have observed no association between cluster density and read quality within the current standard working range of cluster density.

Examples of good and bad datasets can be downloaded from the project website http://solexaqa.sourceforge.net/.

### Effects of dynamic read trimming

To determine the benefits of dynamic trimming on downstream applications, we briefly explored one such application: the effects of read trimming on *de novo *assembly. Here, miscalled bases will produce *k*-mers (*i.e.*, sequences with a word length of *k*) that do not reflect the true genome sequence. These false *k*-mers unnecessarily complicate the de Bruijn graph, and might be expected to produce poorer assemblies. To test this, we examined a dataset containing the genomes of 20 bacterial isolates from two closely related species, *Campylobacter coli *and *C. jejuni*, which were sequenced as indexed (*i.e*., individually bar-coded) samples using 50-bp single-end sequencing on an Illumina Genome Analyzer II. These data were pre-processed with Illumina's proprietary pipeline software (version 1.4), which yielded ~3 million reads per genome (~90-fold average nucleotide coverage). Individual reads were either trimmed dynamically using DynamicTrim or submitted unaltered to Velvet (version 0.7.60) [10] for *de novo *assembly. In both cases, we explored a *k*-mer parameter sweep of 17 to 49, with a fixed coverage cutoff of 5, and expected *k*-mer coverage inferred from the number of reads used and the expected genome size. *De novo *assemblies were summarized using N_50 _and the maximum contig size.

**Figure 4:**
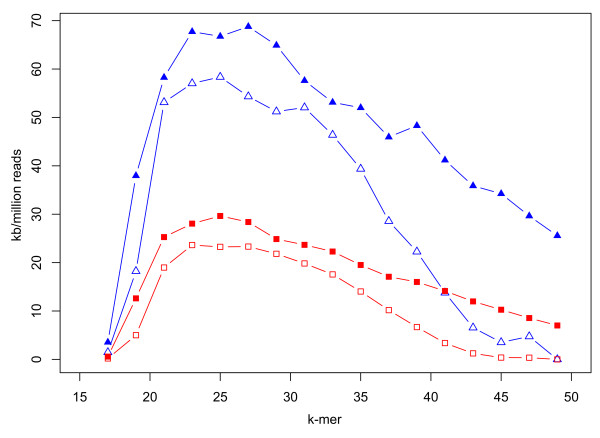
**Effect of dynamically trimmed *versus *untrimmed reads on *de novo *assembly with the Velvet assembler**. Dynamically trimmed reads (solid symbols) relative to untrimmed reads (open symbols) yield improved N_50 _values (red squares) and maximum contig sizes (blue triangles). Summary statistics were averaged across *de novo *assemblies for 20 isolates of *Campylobacter coli *and *C. jejuni*, and normalized by the total number of reads employed in each assembly.

Mean values of these summary statistics, normalized by the number of reads used in each assembly, are plotted in Figure 4. On average, dynamic read trimming produced larger N_50 _and maximum contig sizes. Importantly, fewer trimmed reads were used to produce these assemblies, and the genome sequences therefore assembled much more quickly and required fewer computational resources. As expected, the benefits of dynamic trimming are reduced for extremely good datasets - if data quality is high, there is little difference between trimmed and untrimmed datasets.

We have also encountered instances of run- and species-specific assembly effects. In our experience, the same library preparation sequenced on the same machine on different occasions can produce data of quite different quality. We have also noticed that read quality often differs between species, even where sample quality is similar and samples are run - as indexed reads - in exactly the same flow cell lane. We suspect that the specific characteristics of individual genomes, such as G+C content and repeat prevalence, have important effects on sequence data quality. These anecdotes illustrate the idiosyncratic nature of individual datasets and emphasize the need to test a range of assembly algorithms and data manipulations (including no read trimming) before settling on a final assembly. Generally speaking, however, we found that dynamic trimming of reads produced better *de novo *assemblies of several *Campylobacter *genomes using the Velvet assembler, and we have noted similar improvements in other downstream applications for a range of prokaryotic and eukaryotic datasets. For instance, dynamically trimmed reads appear to improve the signal-to-noise ratio substantially when calling single nucleotide polymorphisms (SNPs).

## Conclusions

The SolexaQA package produces tabular and graphical summaries of data quality for sequence datasets generated with Illumina's second-generation sequencing machines. This package aims, firstly, to create standardized diagnostic information to help identify low-quality data rapidly and easily, and secondly, to provide a dynamic trimming function to manipulate sequence data at the level of individual reads. The SolexaQA package processes even large files within minutes, and produces trimmed datasets that yield significant improvements in downstream analyses, including SNP calling and *de novo *sequence assembly.

## Availability and Requirements

Project name: SolexaQA

Project home page: http://solexaqa.sourceforge.net/

Operating system(s): Platform independent with primary UNIX support

Other requirements: Requires Perl http://www.perl.org/, R http://www.r-project.org/, matrix2png http://www.bioinformatics.ubc.ca/matrix2png/, and the GD graphics library http://www.libgd.org/.

Programming languages: Perl and R

License: GNU GPL version 3 or later

## Authors' contributions

MPC and PJB proposed the algorithm. MPC designed the code. MPC and DAP implemented the software. MPC, DAP and PJB performed the analyses. MPC wrote the paper. All authors have read and approved the final manuscript.
